# The Epidemiological Signature of Pathogen Populations That Vary in the Relationship between Free-Living Parasite Survival and Virulence

**DOI:** 10.3390/v12091055

**Published:** 2020-09-22

**Authors:** Lourdes M. Gomez, Victor A. Meszaros, Wendy C. Turner, C. Brandon Ogbunugafor

**Affiliations:** 1 Department of Ecology and Evolutionary Biology, Yale University, New Haven, CT 06520, USA; lourdes.gomez@yale.edu; 2 Department of Ecology and Evolutionary Biology, Brown University, Providence, RI 02912, USA; victor_meszaros@brown.edu; 3 Department of Biological Sciences, University at Albany–State University of New York, Albany, NY 12222, USA; wcturner@albany.edu

**Keywords:** virulence, free-living survival, disease dynamics, evolution of infectious disease

## Abstract

The relationship between parasite virulence and transmission is a pillar of evolutionary theory that has implications for public health. Part of this canon involves the idea that virulence and free-living survival (a key component of transmission) may have different relationships in different host–parasite systems. Most examinations of the evolution of virulence-transmission relationships—Theoretical or empirical in nature—Tend to focus on the evolution of virulence, with transmission being a secondary consideration. Even within transmission studies, the focus on free-living survival is a smaller subset, though recent studies have examined its importance in the ecology of infectious diseases. Few studies have examined the epidemic-scale consequences of variation in survival across different virulence–survival relationships. In this study, we utilize a mathematical model motivated by aspects of severe acute respiratory syndrome coronavirus 2 (SARS-CoV-2) natural history to investigate how evolutionary changes in survival may influence several aspects of disease dynamics at the epidemiological scale. Across virulence–survival relationships (where these traits are either positively or negatively correlated), we found that small changes (5% above and below the nominal value) in survival can have a meaningful effect on certain outbreak features, including *R*_0_, and on the size of the infectious peak in the population. These results highlight the importance of properly understanding the mechanistic relationship between virulence and parasite survival, as the evolution of increased survival across different relationships with virulence may have considerably different epidemiological signatures.

## 1. Introduction

Interactions between the life history of a pathogen and the environment in which it is embedded drive the evolution of virulence. These interactions thus dictate both the experience of disease at the individual host level and the shape of disease dynamics in host populations [1,2]. The nature of the interaction between virulence and transmission has been the object of both theoretical and empirical examination [2,3,4,5,6,7,8]. Free-living survival, here defined as the ability of a pathogen to persist outside of its host, is one of many transmission life-history traits associated with virulence. The relationship between the two varies between host–pathogen types and different environments [4,8,9,10].

Several hypotheses serve as the canon in the evolution of virulence, theorizing its relationship with transmission traits. The Curse of the Pharaoh hypothesis—Named after a tale about a mythical curse that torments individuals who dig up tombs of Egyptian pharaohs [11]—Suggests that, if a parasite has high free-living survival, then it is far less dependent on its host for transmission and, consequently, will have no evolutionary incentive to decrease virulence [2,4,12]. The potential negative fitness consequences of killing hosts rapidly (being highly virulent) can be counteracted by persisting in the environment until the arrival of new susceptible hosts. Any presumptive selection on beneficence may be relaxed: parasites can detrimentally affect the health of hosts at no cost to transmission because most of their life cycle is spent outside of a host. Previous studies support a positive correlation between free-living survival and mortality per infection (a common proxy for virulence) [13].

Alternatively, the “tradeoff” hypothesis suggests that there is some intermediate level of parasite virulence [3,6,14] that is optimal for a given setting. In this scenario, too high a virulence kills the host and parasite and too low a virulence leads to failure to transmit. Applying this hypothesis specifically to free-living survival would suggest that selection for increased free-living survival should come at the expense of virulence (producing a pathogen that is less harmful to the host). Mechanistically, as a consequence of increased adaptation to a nonhost environment, a virus may be less fit to replicate inside a host [9,15]. For example, a more robust viral capsid may help to survive harsh environmental conditions but may make it more difficult to package RNA/DNA [15]. More generally, the tradeoff hypothesis can be framed in the context of a life-history tradeoff: investment in certain parts of the life cycle often comes at the expense of others [2,16].

Theoretical studies have explored varying evolutionary relationships between heightened virulence and extreme pathogen longevity [4,5,12,17,18,19]. One critical component of these studies revolves around whether virulence evolves independently of free-living survival. For example, some models have argued [4] that pathogen virulence is independent of survival under a set of conditions: when the host–pathogen system is at an equilibrium (evolutionary and ecological), if host density fluctuates around an equilibrium, or if turnover of the infected host population is fast relative to the pathogen in the environment. However, if the host–pathogen system is at disequilibrium and if the dynamics of propagules in the environment are fast compared to the dynamics of infected hosts, then virulence is, as hypothesized, an increasing function of propagule survival [4]. Kamo and Boots [17] examined this hypothesis by incorporating a spatial structure in the environment using a cellular, automata model and found that, if virulence evolution is independent of transmission, then long-lived infective stages select for higher virulence. However, if there is a tradeoff between virulence and transmission, there is no evidence for the Curse of the Pharaoh hypothesis, and in fact, higher virulence may be selected for by shorter rather than long-lived infectious stages. Further, the evolution of high virulence does not have to occur solely through a transmission–virulence tradeoff. Day [18] demonstrated how pathogens can evolve high virulence and even select for traits to kill the host (e.g., toxins) if pathogen transmission and reproductive success are decoupled. These studies emphasized the context-dependence of virulence–survival relationships. Understanding where in the relationship between virulence and survival a given pathogen population exists may allow one to understand how virus evolution will manifest at the level of epidemics.

In this study, we examine the epidemic consequences of different virulence–survival relationships—Positive and negative correlation—In a viral disease with an environmental transmission component. In order to measure how pathogen survival influences disease dynamics, we included an environmental compartment in our model, which represents contaminated environments that act as a reservoir for persisting pathogens, causing disease spread when they come in contact with susceptible individuals (infection via “fomites”) [20,21].

We find that the identity of the virulence–free-living survival relationship (e.g., positive vs. negative) has distinct implications for how an epidemic will unfold. Some, but not all, features of an outbreak are dramatically influenced by the nature of the underlying virulence–survival relationship. This indicates that signatures for evolution (adaptive or other) in a pathogen population will manifest more conspicuously in certain features of an outbreak. We reflect on these findings in light of their theoretical implications on the evolution and ecology of infectious disease and for their potential utility in public health interventions.

## 2. Materials and Methods

### 2.1. Model Motivation and Application

The mathematical model explored in this study is adapted from a recent one developed to investigate environmental transmission of SARS-CoV-2 during the early-stage outbreak dynamics of coronavirus disease 2019 (COVID-19), with parameter values based on fits to actual country outbreak data [22]. In this study, we utilize this model to examine questions about the evolution of free-living survival. While the phenomenon we examine is a very relevant one that manifests in the real world, we want to emphasize that none of the methods or results in this study are intended to be applied to the current COVID-19 pandemic (as of September, 2020). This study is an attempt at responsible theoretical biology, with data-informed models and inferences that are germane to the natural world. However, neither do we support the extrapolation of these findings to any particular aspect of COVID-19 nor should they inform a policy or intervention. The model applies to a number of scenarios that include outbreaks in a naïve host population. This describes situations such as the evolution of novel viral lineages, viral spillover events, or host shifts, where a virus with a preexisting relationship between virulence and survival emerges in a population of new hosts. Another such scenario where this model applies is one where a virus has already emerged but evolves in a subpopulation in the novel hosts before a migration event of some kind introduces the evolved virus population to a fully susceptible population of hosts.

### 2.2. Model Description

The model is implemented via a set of ordinary differential equations, defined by Equations (1)–(6). It implements viral free-living survival via the “Waterborne Abiotic or other Indirect Transmission (WAIT)” modelling framework, coupling individuals and the pathogen within the environment [23,24].

Within the model, the *β_w_* term allows for individuals to become infected via viral pathogen deposited in the environment and terms *𝜎_A_* and *𝜎_I_* allow asymptomatic and symptomatic individuals to deposit pathogens into the environment, respectively. Adapted from the more traditional SEIR (susceptible-exposed-infected-recovered) model, the SEAIR-W (susceptible-exposed-asymptomatic-infected-recovered-WAIT) model interrogates the consequences of the two hypotheses outlined above while representing the dynamics of a very relevant disease system (SARS-CoV-2) that includes an asymptomatic infectious population. While the importance of asymptomatic transmission was debated early in the pandemic, many studies have affirmed its role in the spread of disease [25,26,27]. Though environmental transmission of SARS-CoV-2 remains a controversial topic, it is plausible that asymptomatic individuals may spread disease through frequent contact with the environment, thus increasing the proportion of virus that is free-living [28]. We acknowledge that mathematical models of epidemics can be limited by “identifiability,” which can obfuscate the relative importance of some routes of transmission. In models that have both indirect and direct routes of transmission, it can be very difficult to conclude that one route is predominant [29,30,31].

(1) $$\frac{dS}{dt}=\mu \left(N-S\right)-\left(\frac{\beta_{A}A+\beta_{I}I}{N}+\beta_{W}W\right)S$$

(2) $$\frac{dE}{dt}=\left(\frac{\beta_{A}A+\beta_{I}I}{N}+\beta_{W}W\right)S-\left(\varepsilon +\mu \right)E$$

(3) $$\frac{dA}{dt}=\varepsilon E-\left(\omega +\mu \right)A$$

(4) $$\frac{dI}{dt}=\left(1-p\right)\omega A-\left(v+\mu_{I}\right)I$$

(5) $$\frac{dR}{dt}=p\omega A+vI-\mu R$$

(6) $$\frac{dW}{dt}=\left(\frac{\sigma_{A}A+\sigma_{I}I}{N}\right)\left(1-W\right)-kW$$

**Figure 1:**
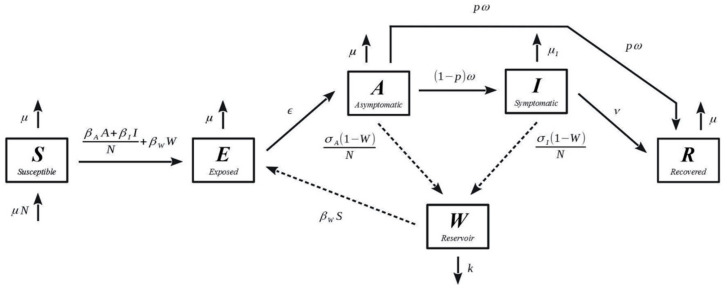
Compartmental diagram of the SEAIR-W (susceptible-exposed-asymptomatic-infected-recovered) version of a-WAIT (Waterborne Abiotic or other Indirect Transmission)) model: this is based on a previously developed mathematical model used to interrogate environmental transmission of SARS-CoV-2 (see [22]).

Figure 1 depicts the compartmental diagram for the model. The direction of the arrows corresponds to the flow of the individuals and the pathogen through the system. Note that individuals can move directly from the asymptomatically infected compartment to the recovered compartment (bypassing the symptomatic compartment) via what we call a “mild track”. The dashed arrows represent WAIT coupling to the environment. The model is inspired by one developed to interrogate environmental transmission of SARS-CoV-2 [22].

### 2.3. Simulations of Outbreaks

The system was numerically integrated using the “odeint” solver in the Scipy 1.4—Python scientific computation suite [32]. The simulations track the populations for each of the compartments listed in Figure 1. Each model run occurred over 250 days, which amounts to over 8 months of the epidemic or 5× the peak of the outbreak. This length of time is consistent with the antecedent SARS-CoV-2 model [22], long enough for the dynamics of the system to manifest. Note however that, for this study, we are especially interested in the early window of an outbreak: the first 30 days. We focus on this window because this is the time frame that best captures the underlying physics of an epidemic, as 30 days is often before populations are able to adjust their individual behaviors. The code constructed for the analysis in this study is publicly available on github: https://github.com/OgPlexus/Pharaohlocks.

### 2.4. Population Definitions and Parameter Values

Table 1 outlines the definitions of each population and provides the initial population values used for all simulations conducted in this study. The nominal parameter values used are defined in Table 2. The initial values are drawn from the aforementioned COVID-19 outbreak study, derived from empirical findings and country-level outbreak data [22].

### 2.5. Virulence Definition

In this study, we define virulence as the capacity to cause a disease. In order to measure it, we utilize a set of parameters that uniformly increase the rate or probability of causing symptomatic disease or the severity of those symptoms (including death). Our definition is more comprehensive than many other models of parasite virulence (e.g., [4,13]), which tend to focus on a single aspect of the natural history of disease associated with harm to a host (e.g., the fitness consequences of an infection on the host population or the case fatality rate). Instead of having to justify a definition built around a single term (e.g., the term associated with fatality), we took a collective approach to defining virulence through all terms that foment the viral-induced onset of symptomatic disease and death. This definition allows for the reality of pleiotropic effects in viral pathogens, where adaptations can have multiple effects on the natural history of disease [2,33]. Our definition of virulence emphasizes terms that influence host wellness and/or are symptoms of disease. The iteration of virulence used in this study also undermines the potential for overly weighting only one or a small number of parameters under a large umbrella of virulence. Because so many varying definitions exist for virulence, we have also performed calculations according to a different definition of virulence, one that exclusively considers terms that have a detrimental direct effect on the host and neither of the terms that reflect symptoms of severe disease (*𝜎_a_* and *𝜎_I_*). These calculations can be found in the Supplementary Materials.

The collection of parameters that we use to define virulence are as follows: the infected population death rate (*𝜇_I_*), the incubation period of SARS-CoV-2 (*𝜂*), the rate of transfer from asymptomatic to symptomatic (1/*⍵*), the infected population recovery rate (*ν*), the percent of individuals that move from the asymptomatic to the recovered compartment without showing symptoms (the “mild” recovery track, *p*), the contact rate of people with people × the transmission probability of people to people by an asymptomatic individual (*β_A_*), the contact rate of people with people × the transmission probability of people to people by an asymptomatically infected person (*β_I_*), the contact rate of people with the environment × the probability of shedding by an asymptomatic individual to the environmental (*𝜎_A_*), the contact rate of people with the environment × the probability of symptomatically infected individuals shedding in the environment (*𝜎_I_*), and the average number of days before infection (1/*ε*).

Table 3 outlines the direction in which each of the virulence-associated parameters are modulated as virulence decreases or increases. An up arrow (↑) indicates the parameter increases (by an equivalent percent) when the percent virulence is changed. A down arrow (↓) indicates the parameter decreases (by an equivalent percent) when the percent change in virulence is applied. Changes in virulence are then defined, in this study, as an equivalent uniform (percent) change in each of the parameters listed above. For the purposes of our study, we modify virulence by changing all parameters associated with virulence by 5%. One could also disambiguate virulence into changes in individual subcomponents; however, that is not the focus of this current study.

### 2.6. Survival Definition

Survival is defined as the set of parameters that, when uniformly modulated, increases the pathogen’s probability of surviving the outside environment and successfully infecting a new host [2]. In our model, this includes both the waning virus rate in the environment (*k*) and the contact rate of an individual with the environment × the transmission probability of the environment to people (*β_w_*). Table 4 outlines the direction (increasing or decreasing) in which these parameters are modulated when survival is decreased or increased. Within both models, a (percent) change in survival is defined as an equivalent uniform (percent) change in the survival parameters.

Throughout this study, the impact of changes in virulence and survival (and the relationship between these traits) are assessed with respect to the following four epidemic metrics: the number of infected individuals (asymptomatic and symptomatic) at the maximum (when the outbreak is at its most severe), the rate at which the peak infected population is reached (t_max_^−1^), the total infected population after 30 days, and the basic reproductive ratio (*R*_0_). Importantly, among these signatures, the basic reproductive ratio is the most frequently used in epidemiology and benefits from familiarity and mathematical formalism (see Section 2.7). The other signatures are determined through simulations of an epidemic for a given set of parameter values. Nonetheless, this study’s inclusion of multiple features of the epidemic allows us to examine how variation in virus life-history traits may influence different aspects of an epidemic in peculiar ways.

### 2.7. Basic Reproductive Ratio

Equations (7)–(9) give the analytic expression of the basic reproductive ratio (*R*_0_) for the model used in this study. This expression for *R*_0_ can be deconstructed into two components. Equation (8) only contains parameters associated with person to person transmission (*R_p_*), while Equation (9) solely contains parameters associated with transmission from the environment (*R_e_*). In the Supplementary Materials, we provide additional information on these terms and their derivations. Applying the parameters values in Table 2, the numerical value of the basic reproductive ratio is given as *R*_0_ ~ 2.82.

(7) $$R_{0}=\frac{R_{p}\sqrt{R_{p}^{2}+4R_{e}^{2}}}{2}$$

(8) $$R_{p}=\frac{\varepsilon \left(\beta_{A}\left(\mu_{I}+v\right)+\beta_{I}\left(1-p\right)\omega \right)}{\left(\mu +\varepsilon \right)\left(\mu +\omega \right)\left(\mu_{I}+v\right)}$$

(9) $$R_{e}^{2}=\frac{\varepsilon \beta_{W}\left(\sigma_{A}\left(\mu_{I}+v\right)+\sigma_{I}\left(1-p\right)\omega \right)}{k\left(\mu +\varepsilon \right)\left(\mu +\omega \right)\left(\mu_{I}+v\right)}$$

## 3. Results

### 3.1. Model Sensitivity Analysis

**Figure 2:**
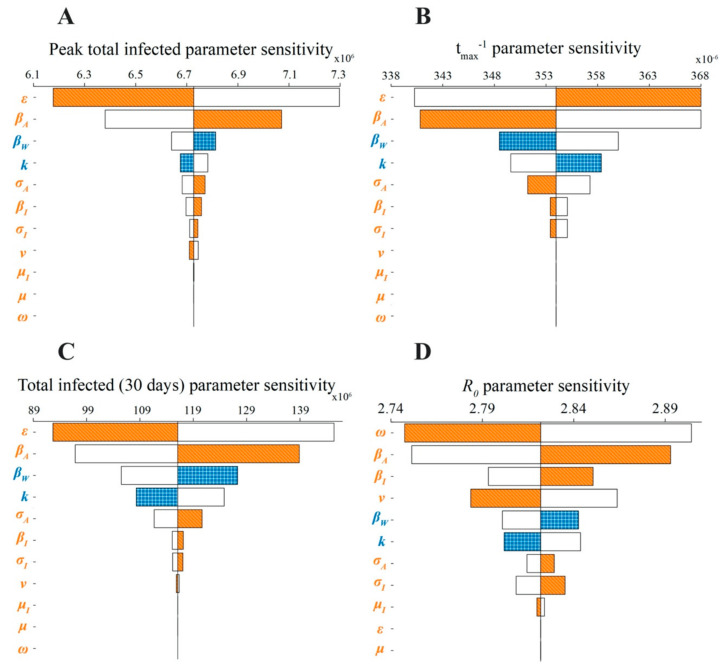
Tornado plot showing the sensitivity of epidemic properties to individual parameter changes: (**A**) the number of infected individuals (asymptomatic and symptomatic) at the epidemic peak; (**B**) the rate at which the epidemic peak is reached, t_max_^−1^; (**C**) the total infected population after 30 days; and (**D**) the basic reproductive ratio (*R*_0_). Filled bars indicate the value of the epidemic feature when the associated parameter is increased by 5.0% from its nominal value. White bars indicate the value of a feature when the associated parameter is decreased by 5.0%. Blue coloring with checkered patterning indicates a parameter associated with survival, and orange coloring with lined patterning indicates a parameter associated with virulence.

Figure 2 depicts a tornado plot that communicates the sensitivity of the model to permutations in parameters. Across features, the model is most sensitive to parameters that are considered virulence-associated (Table 3) and is relatively less sensitive to survival-associated parameters (Table 4). Similar to other features, *R*_0_ (Figure 2D) of the model is most sensitive to the parameters *⍵*, *β_A_*, and *ν*. The sensitivity of *R*_0_ to changes in *⍵* reflects the importance of the rate of conversion to the symptomatic state on model dynamics. In addition, *β_A_* has a very important influence on the model, consistent with other findings for COVID-19 that have emphasized the importance of asymptomatic transmission in disease spread [25,26,27].

### 3.2. Illustrative Dynamics of Model System

**Figure 3:**
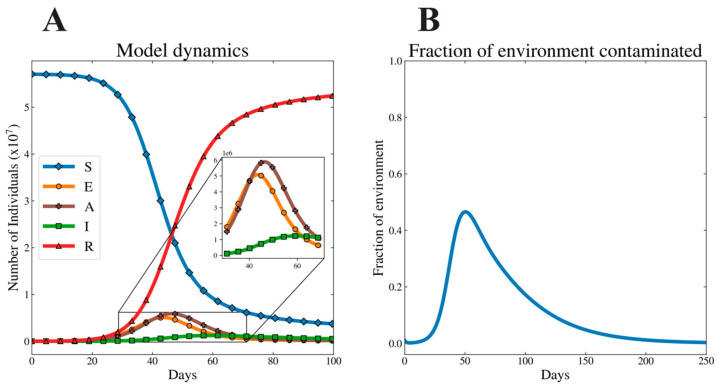
Sample dynamics for the model system: (**A**) the dynamics for all host compartments within the model and (**B**) the fraction of environmental reservoirs in a setting that are contaminated with infectious virus.

Based on the parameter values in Table 2, Figure 3A demonstrates the base dynamics of the model playing out over the first 100 days while Figure 3B shows the dynamics within the environment over the course of 250 days. In these dynamics, the population begins to be fixed for susceptible hosts. The disease dynamics manifest in the shapes of the curves corresponding to exposed, asymptomatic, and symptomatic individuals. Note the long tail of the curve corresponding to contamination by the environment. The environment remains infectious even after the infected populations have declined in number. The length and shape of this tail are influenced by the free-living survival of the virus.

### 3.3. The Epidemic Consequences of Varying Virulence and Survival

In the next analysis, we examine the epidemic consequences of varying traits associated with survival and virulence. One can consider this as a scenario where we compare the endpoints of evolution of different virus populations (corresponding to combinations of values of survival and virulence) and calculating how these evolved populations manifest in epidemic terms.

**Figure 4:**
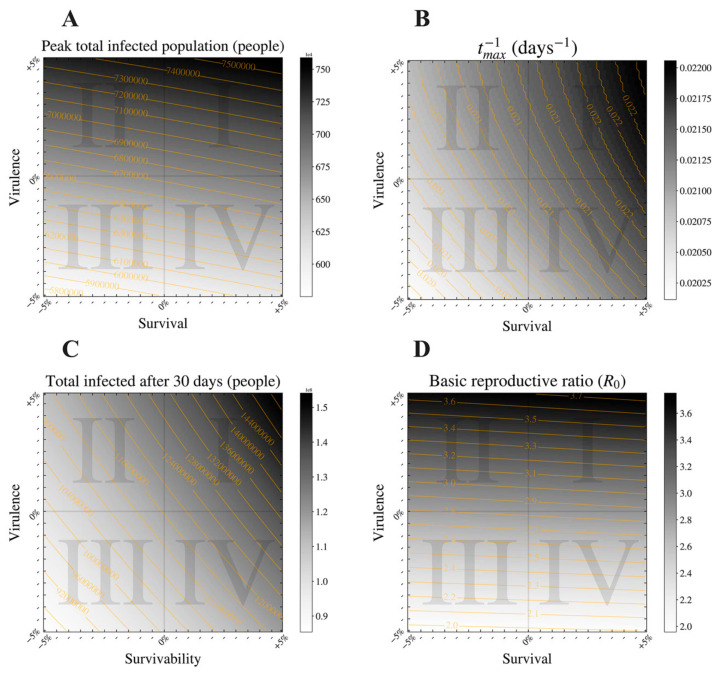
Heatmap describing the impact of varying virus virulence and survival trait values assessed across four key epidemic metrics: these heatmaps express the change in (**A**) the number of infected individuals (asymptomatic and symptomatic) at the epidemic peak; (**B**) the rate at which the epidemic peak is reached, t_max_^−1^; (**C**) the total infected population after 30 days; and (**D**) the basic reproductive ratio (*R*_0_) when virulence and survival are modulated by ±5% within the model. Contour lines are available for clarity.

In Figure 4, we observe how dynamics of the outbreak are influenced across a space of combinations of traits altering virulence (see Table 3 for a list of virulence-associated parameters) and survival (see Table 4 for a list of survival-associated parameters), changed by ±5% (10% overall). In Figure 4D, we demonstrate how changes in virulence and free-living survival traits influence *R*_0_, with variation in virulence-related traits having the largest effect on *R*_0_. Of note is how the range in *R*_0_ values varies widely across virulence–survival values, from nearly 2.0 to 3.7 (Figure 4D).

### 3.4. Implications of Virulence–Survival Relationships at Their Relative Extremes

Having observed how outbreak dynamics are influenced by variation in traits that alter virulence–survival phenotypes, we then examined how each outbreak metric is influenced by the extreme (±5%) values of the trait combinations considered. Specifically, we assess how a change in pathogen survival affects outbreak dynamics, based on two expected relationships between survival and virulence traits.

### 3.5. Positive Correlation Between Survival and Virulence

**Figure 5:**
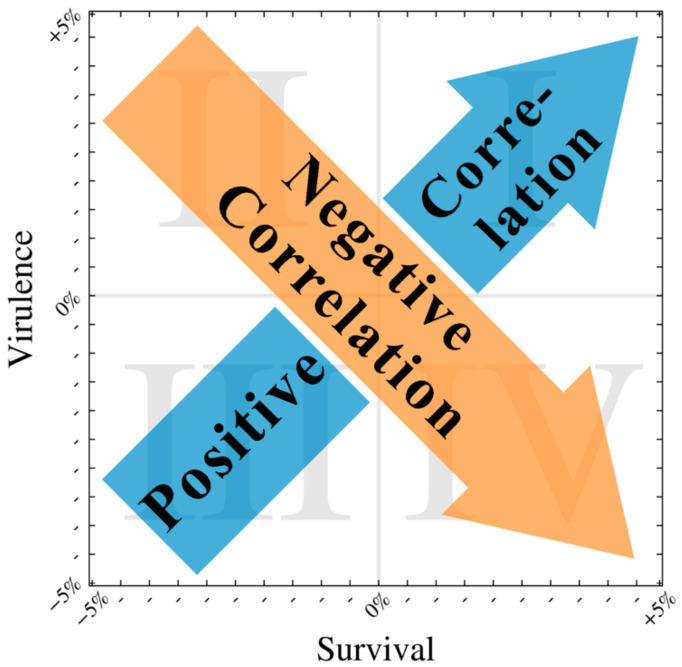
The expected effect of increasing survival on virulence for the two correlation models considered: here, we present a schematic of how the different hypotheses for the relationship between virulence and survival manifest on a map with a structure similar to the heat maps shown in Figure 4. The directions of the arrows depict how increasing survival would affect virulence under the two hypotheses: the blue arrow indicates the flow of an increasing positive correlation dynamic, while the direction of the orange arrow indicates an increasing negative correlation dynamic.

In a positive correlation scenario, high values for survival would be associated with high values for virulence [4,13]. Because the correlations we observe are often not exactly linear, we utilize quadrants to express a trend, allowing for some variance around the expected “line”. In Figure 5, the positive correlation scenario can be represented by combinations of virulence and survival residing in quadrants I and III.

**Figure 6:**
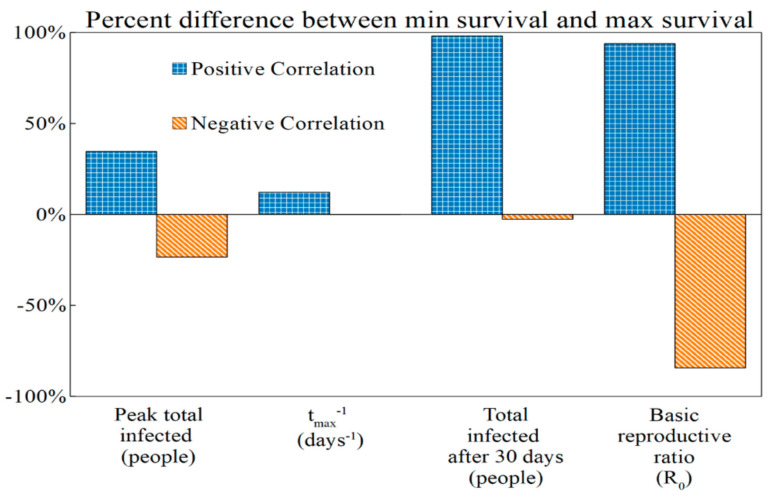
The percent change in SEAIR-W outbreak metrics as survival increases from −5% to +5% for different virulence–survival relationships (positive correlation and negative correlation). For each metric analyzed, we present the percent difference between the minimum and maximum survival values given the two hypotheses tested: (i) positive correlation between survival and virulence (comparing low virulence/low survival to high virulence/high survival) and (ii) negative correlation (high virulence/low survival to high survival/low virulence). The bars here correspond to the values (percent) in the third columns of Table 5 and Table 6, which denote the differences between the minimum and maximum values.

If host–pathogen evolution proceeds according to a positive correlation scenario, all outbreak metrics would show an increase in severity as both survival and virulence increase. Across the range of variation in virulence and survival traits considered (5% above and below the nominal value), the peak number of infected individuals increases by approximately 35%, the rate at which the peak is reached increases by approximately 16%, the total number of infected individuals after 30 days increases by approximately 98%, and *R*_0_ increases by approximately 94% (Figure 6 and Table 5).

### 3.6. Negative Correlation Between Survival and Virulence

In a negative correlation scenario, high values for survival would be associated with low values for virulence [2,9,15] and a low peak in total infected population. Pathogens with a life history that exhibits negative virulence–survival associations would likely appear in quadrants II and IV in Figure 5.

Under negative correlation, outbreak severity decreases as survival increases. Across the measured range of variation in virulence–survival traits, the peak number of infected individuals decreases by approximately 23%, the rate at which the epidemic peak is reached decreases by 0.15%, the total number of infected individuals decreases by 3%, and *R*_0_ decreases by approximately 84% (Figure 6 and Table 6). Across all metrics considered, the effects of increased viral survival on outbreak dynamics is more extreme under the positive correlation than the negative correlation scenarios (Figure 6).

### 3.7. Dynamics of Epidemics at Extreme Values for Virus Free-Living Survival

**Figure 7:**
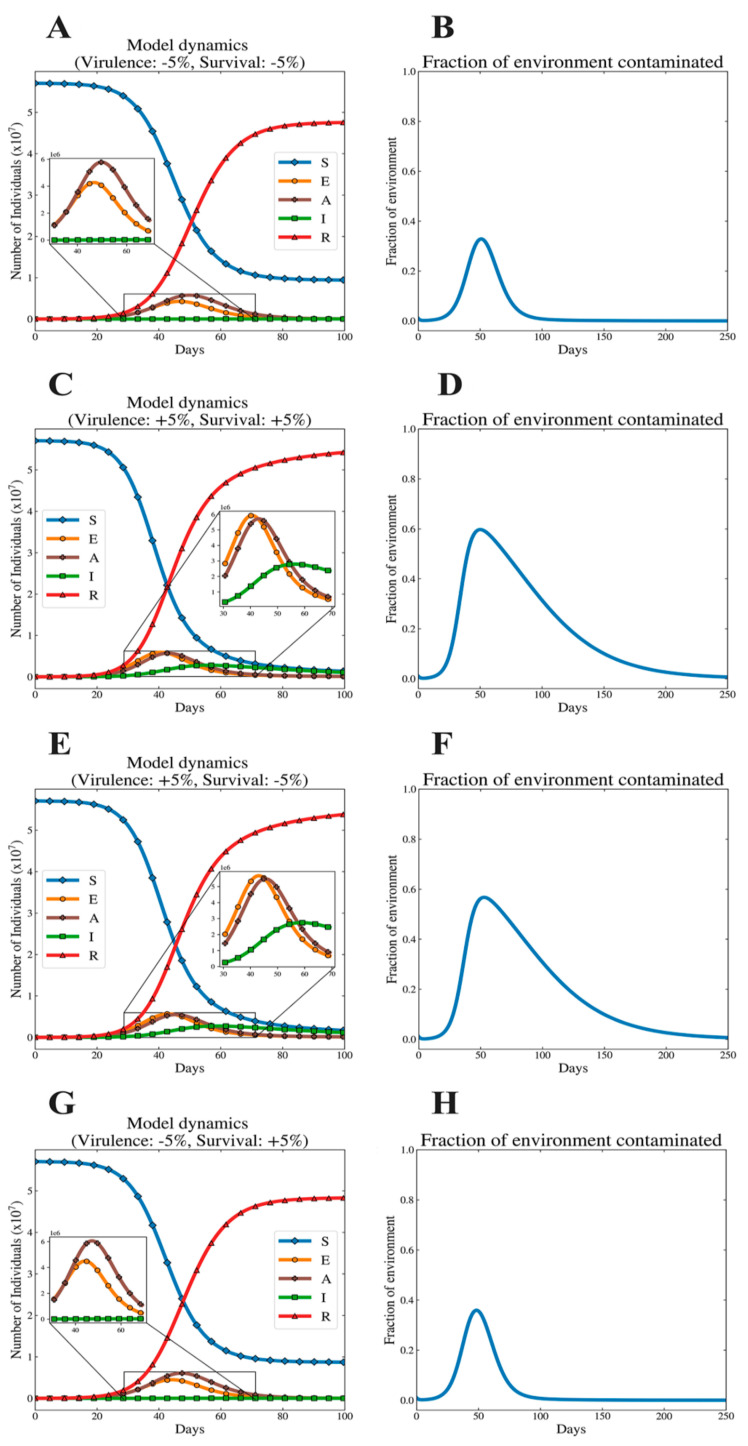
Virus outbreak dynamics for the extreme values of virulence and free-living survival considered for the two different relationships (positive or negative) between virulence and survival traits: these plots are similar to the illustrative dynamics in Figure 1. Here, we observe the dynamics of disease corresponding to the extreme values presented in Table 5 and Table 6. Subfigures (**A**,**C**,**E**,**G**) depict disease dynamics, and (**B**,**D**,**F**,**H**) depict the dynamics of contaminated environments. (**A**–**D**) correspond to the parameter values considered for the positive correlation scenario, while (**E**–**H**) correspond to the negative correlation scenario.

In Figure 7, we observe the disease dynamics at extreme values for survival and the dynamics corresponding to the fraction of the environment that is contaminated with infectious virus. Consistent with the data represented in Figure 6, we observe that minimum and maximum simulations differ more substantially for extreme survival scenarios in the positive correlation scenario than for the negative correlation scenario.

The feature of different outbreaks that varies most ostensibly between the correlation scenarios is the time needed to reach the peak number of infected individuals. In positive correlation simulations, one can observe that the low virulence, low survival scenario (Figure 7A,B) takes longer to reach the peak number of infected individuals. Most notably, however, the low virulence, low survival setting has a far smaller peak of environmental contamination and shorter tail relative to its high virulence, high survival counterpart (Figure 7D). Similarly, intriguing findings exist in the comparison between the simulation sets corresponding to extremes in the negative correlation setting (Figure 7E–H). Especially notable is the difference in the length of the tail of the environmental contamination for the high virulence, low survival combination (Figure 7F) vs. the low virulence, low survival combination variant (Figure 7H). The explanation is that, in this model, higher virulence influences (among many other things) the rate at which the virus is shed into the environment from either the asymptomatic (*𝜎_A_*) or symptomatic (*𝜎_I_*) host. We observe how the high virulence, low survival simulation (Figure 7E) features a symptomatic peak that is larger in size and is prolonged relative to the lower virulence counterpart (Figure 7G). This relatively large symptomatic population sheds infectious virus into the environment for a longer period of time, contributing to the long tail of contaminated environments observed in Figure 5F.

## 4. Discussion

The virulence–survival relationship drives the consequences of virus evolution on the trajectory of an outbreak. In this study, we examined how different virulence–survival relationships may dictate different features of outbreaks at the endpoints of evolution (according to the positive or negative correlation scenarios). When the parameter space for virulence and survival is mapped, we find that certain outbreak metrics are more sensitive to change in free-living survival and virulence than others and that the nature of this sensitivity differs depending on whether survival and virulence are positively or negatively correlated.

For the positive correlation scenario, when free-living survival varies between 5% below and above the nominal value, we observed a dramatic change in the total number of infected individuals in the first 30 days (98% increase from minimum survival to maximum survival; Table 5), and *R*_0_ nearly doubles (94% increase; Table 5). These two traits are, of course, connected: the theoretical construction of the *R*_0_ metric specifically applies to settings where a pathogen spreads in a population of susceptible hosts [34,35], an early window that is captured in the first 30 days.

When survival and virulence are negatively correlated, different outbreak dynamics emerge: while the *R*_0_ difference between minimum and maximum survival is significant (approximately 84% decrease), the total number of infected individuals only changes by roughly 3% (Table 6). This large difference between *R*_0_ at higher and lower survival values also does not translate to a difference in the total number of infected individuals in the first 30 days of an infection (the early outbreak window). In a scenario where survival and virulence are negatively correlated, a highly virulent and less virulent virus population can have similar signatures on a population with respect to the number of infected individuals in the first month. Thus, simply measuring the number of infected individuals in the first month of an outbreak is unlikely to reveal whether a pathogen population has undergone adaptive evolution or has evolved in a manner that meaningfully influences the natural history of disease.

Notably, for scenarios where survival and virulence are both positively and negatively correlated, the time that it takes for an epidemic to reach its maximum number of infected individuals changes little across extreme values of survival (12% in the positive correlation scenario; 0.15% in the negative correlation scenario; see Table 5 and Table 6). That is, the time that it takes for an epidemic to reach its peak (however high) is not especially sensitive to evolution in virulence or survival.

### Practical Implications for the Understanding of Outbreaks Caused by Emerging Viruses

That different features of an outbreak are differentially influenced by the endpoints of viral life-history evolution highlights how epidemiology should continue to consider principles in the evolution and ecology of infectious disease in its analyses and predictions. As not all features of an epidemic are going to be equally reliable signatures of virus evolution, we should carefully consider the data on how the dynamics of an epidemic change when making inferences about whether a pathogen population is essentially different from prior iterations (e.g., prior outbreaks of the same virus type). The results of this study suggest that carefully constructed, mechanistically sound models of epidemics are important, both for capturing the dynamics of an outbreak and for abetting our efforts to understand how evolution of survival and virulence influences disease dynamics.

For example, the potential for adaptive evolution of SARS-CoV-2 has emerged as a possible explanation for different COVID-19 dynamics in different countries. We suggest that such interpretations should be considered with caution and that they require very specific types of evidence to support them. As of 1 July 2020, any conclusion that widespread SARS-CoV-2 evolution is an explanation for variation in disease patterns across settings (space and/or time) is premature.

The practical process of interpreting the evolutionary consequences of signals of virus evolution should encompass several discrete steps. Firstly, we should determine whether molecular signatures exist for adaptive evolution. Adaptive evolution would manifest in observable differences in genotype and phenotype and, perhaps, in the natural history of disease. Secondly, we should aim to attain knowledge of the underlying mechanistic relationship between survival and virulence. This knowledge is not necessarily easy to attain (it requires extensive laboratory studies) but would allow added biological insight: we may be able to extrapolate how changes in some traits (e.g., those that compose survival) influence others (e.g., those that influence virulence).

More generally, our findings suggest that the ability to detect the consequences of virus evolution would depend on which feature of an outbreak an epidemiologist measures: from our analysis, *R*_0_ is most impacted by changes in virulence and survival. In addition, the total number of infected individuals in the early window and the size of the infected “peak” would each be impacted most readily by changes in virulence–survival traits. The rate at which the epidemic peak was reached, on the other hand, showed relatively little change as survival increased or between the two correlation scenarios. Consequently, it would not serve as a useful proxy for virus evolution.

While the stochastic, sometimes entropic nature of epidemics renders them very challenging to predict [36], we suggest that canons such as life-history theory and the evolution of virulence provide useful lenses that can aid in our ability to interpret how life-history changes in virus populations will manifest at the epidemiological scale. We propose that, in an age of accumulating genomic and phenotypic data in many pathogen–host systems, we continue to responsibly apply or modify existing theory in order to collate said data into an organized picture for how different components of the host–parasite interaction influence the shape of viral outbreaks of various kinds.

## Figures and Tables

**Table 1:** Definitions and initial values for the populations represented by each compartment: the values here are the averages of the model values across all countries developed in a prior COVID-19 study [22].

Symbols	Value	Units	Definitions
S_0_	57.05 × 10^6^	people	Susceptible individuals
E_0_	66.50	people	Exposed individuals
A_0_	13.30	people	Asymptomatic individuals
I_0_	13.30	people	Symptomatic individuals
Rec_0_	0	people	Recovered individuals
W_0_	1%	unitless	% of viral pathogen in environment

**Table 2:** Definitions for the nominal parameter values used in this study: parameter values were developed from empirical findings and country-level data, as discussed in another study [22].

Symbols	Values	Units	Definitions
𝜇	1/(80.3 × 365)	1/day	Natural death rate (reciprocal of the upper bound of average human lifespan)
𝜇_I_	0.00159	1/day	Infected death rate (natural death rate + disease-induced death rate)
𝜂 = (*⍵* − *ε^−^*^1^*)*	5.5	days	SARS-CoV-2 incubation period
1/*⍵*	𝜂 − *ε*^−1^	days	Expected time in the asymptomatic state
*ν*	0.0305	1/day	Recovery rate (average of 3 to 6 weeks)
*p*	95.6%	percent	Percent that moves along the “mild” recovery track
*k*	0.649	1/day	Waning virus rate in the environment (using the average of all material values, wood, steal, cardboard, and plastic)
*β_a_*	0.550	1/day	Contact rate of people with people × transmission probability of people to people by A-person
*β_I_*	0.491	1/day	Contact rate of people with people × transmission probability of people to people by I-person
*β_W_*	0.031	1/day	Contact rate of person with environment × transmission probability of environment to people
*𝜎_a_*	3.404	1/day	Contact rate of person with environment × probability of shedding by A-people to environment
*𝜎_I_*	13.492	1/day	Contact rate of person with environment) × probability of shedding by I-people to environment
*1/ε*	2.478	days	Average number of days before infectiousness

**Table 3:** Virulence parameters: this is a list of uniformly modulated parameters and the direction in which they change when virulence is increased or decreased. When virulence changes, an up arrow (↑) indicates the parameter is increased (by an equivalent percent) and a down arrow (↓) indicates the parameter is decreased (by an equivalent percent).

Symbols	Definition	Virulence Increased	Virulence Decreased
𝜇_I_	Infected death rate (natural death rate + disease induced death rate)	↑	↓
𝜂 = (*⍵* − *ε*^−1^*)*	SARS-CoV-2 incubation period	↓	↑
1/*⍵*	Expected time in the asymptomatic state	↑	↓
*ν*	Recovery rate (average of 3 to 6 weeks)	↓	↑
*p*	Percent that moves along the “mild” recovery track	↓	↑
*β_A_*	Contact rate of people with people × transmission probability of people to people by A-person	↑	↓
*β_I_*	Contact rate of people with people × transmission probability of people to people by I-person	↑	↓
*𝜎_A_*	Contact rate of person with environment) × (probability of shedding by A-people to environment	↑	↓
*𝜎_I_*	Contact rate of person with environment × probability of shedding by I-people to environment	↑	↓
*1/ε*	Average number of days before infectious	↓	↑

**Table 4:** Survival parameters: this is a list of parameters that are uniformly modulated and the direction in which they change when survival is increased or decreased. An up arrow (↑) indicates the parameter is increased by some percent when the equivalent (percent) change in survival is applied. A down arrow (↓) indicates the parameter is decreased by some percent when the equivalent (percent) change in survival is applied.

Symbols	Definition	Survival Increased	Survival Decreased
*k*	Waning virus rate in environment (using the average of all material values, wood, steal, cardboard, and plastic)	↓	↑
*β_W_*	Contact rate of person with environment × transmission probability of environment to people	↑	↓

**Table 5:** Positive correlation scenario: comparing epidemic metrics under low survival/low virulence versus high survival/high virulence scenarios (as in the positive correlation scenario). For each metric analyzed, these are the heatmap values for the bottom left (at “coordinates” (Vir, Sur) → (−5%, −5%)) and top right (at “coordinates” (Vir, Sur) → (+5%, +5%)) corners.

Epidemic Metric	min Virulence, min Survival	max Virulence, max Survival	% Difference between min Survival and max Survival
**Peak total infected (people)**	5.68 × 10^6^	7.64 × 10^6^	+34.51%
**t_max_^−1^ (days^−1^)**	1.99 × 10^−2^	2.23 × 10^−2^	+12.06%
**Total after 30 days (people)**	8.18 × 10^7^	1.62 × 10^8^	+98.04%
**Basic reproductive ratio (*R*_0_)**	1.95	3.78	+93.84%

**Table 6:** Negative correlation scenario: comparing epidemic metrics under low survival/low virulence versus high survival/high virulence scenarios (as in the negative correlation scenario). For each metric analyzed, these are the global heatmap values for the top left (at “coordinates” (Vir, Sur) → (+5%, −5%)) and bottom right corners (at “coordinates” (Vir, Sur) → (−5%, +5%)).

Epidemic Metric	max Virulence, min Survival	min Virulence, max Survival	% Difference between min Survival and max Survival
**Peak total infected (people)**	7.39 × 10^6^	5.99 × 10^6^	−23.47%
**t_max_^−1^ (days^−1^)**	2.10 × 10^−2^	2.23 × 10^−2^	−0.15%
**Total infected after 30 days (people)**	1.16 × 10^8^	1.13 × 10^8^	−2.68%
**Basic reproductive ratio (*R*_0_)**	3.67	1.99	−84.39%

## Supplementary Materials

The following are available online at https://www.mdpi.com/1999-4915/12/9/1055/s1. Table S1. Fixed parameters, and their references. Table S2. Alternative virulence definition containing only items that directly affect host well-being (fitness). Figure S1. Alternative virulence definition: The impact of varying virus virulence and survival assessed on four key epidemic metrics. Figure S2. Alternative virulence definition: The percent change in SEAIR-W outbreak metrics as survival increases from −5% to +5% for different virulence-survival relationships (positive correlation and negative correlation). Figure S3. Alternative virulence definition: Virus outbreak dynamics for the extreme values of virulence and free-living survival considered for the two hypotheses. Table S3. Alternative virulence definition: Comparing epidemic metrics under low survival/low virulence versus high survival/high virulence scenarios. Table S4. Alternative virulence definition: Comparing epidemic metrics under low survival/low virulence versus high survival/high virulence scenarios (as in the negative correlation scenario). Figure S4. Side by side comparison of the main text virulence definition (A1 → D1) and alternative definition of virulence (A2 → D2). Figure S5. Side by side comparison of the main text virulence definition (left) and alternative definition of virulence (right). Figure S6. Side by side comparison of the main text definition (A_1_ → H_1_) and supplemental definition of virulence (A_2_ → H_2_).
