# Supplementary material for: The Epidemiological Signature of Pathogen Populations That Vary in the Relationship between Free-Living Parasite Survival and Virulence
Source: Viruses. 2020 Sep 22;12(9):1055. doi: 10.3390/v12091055 (PMC7551987; doi:10.3390/v12091055)
Supplement: Supplementary file 1 [file viruses-12-01055-s001.zip › viruses-903183-supplementary.pdf]

viruses

MDPI

Table S1. Fixed parameters, and their references. The mathematical model utilized in this study is derived from an existing one (Ogbunugafor et al., 2020; see main text reference 22). While all references and estimation methods can be found there, here we provide several of the fixed parameters and their references. Alternative definition of virulence. We ran the analysis observed in the main text for a scenario where virulence is only composed of those parameters that directly affect host well-being or fitness. While σ_A and σ_S are traits included in the main text definition reflect traits that don't directly affect host fitness. Instead, they correspond to traits (the shedding of virus) the consequences of infection. In order to examine the results of our analysis using a stricter definition of virulence, we eliminated these from this alternative analysis. Below find the find the virulence definition and a series of analyses that utilize this alternative definition. The differences between the two definitions can be observed in the side by side comparisons (Supplemental figures S4–S6). The conclusions are qualitatively the same.

Symbols	Definitions	References
μ	Natural Death Rate (Reciprocal of the average life expectancy of 17 countries sampled)	S1, S2
μ_I	Infected death rate	S3, S4
η	Incubation Period	S5
$1/\omega$	Expected time in the asymptomatic state	Fitted and dependent on η
ν	Recovery rate (Average of 3 to 6 weeks)	S5
p	Fraction that move along the "mild" recovery track	S6
k	Viral decay rate in environment (using average of all material values, wood, steal, cardboard, plastic)	S7

Table S2. Alternative virulence definition. containing only items that directly affect host well-being (fitness). We ran calculations observed in Figure S1–S3 with this different definition.

Symbols	Definition
μ_I	Infected death rate (Natural death rate + disease induced death rate)
$\eta = (\omega\text{-}\varepsilon^{-1})$	SARS-CoV-2 incubation period
$1/\omega$	Expected time in the asymptomatic state
ν	Recovery rate (Average of 3 to 6 weeks)
p	Percent that move along the "mild" recovery track
β_A	(Contact rate of people with people) × (transmission probability of people to people by A-person)
β_I	(Contact rate of people with people) × (transmission probability of people to people by I-person)
$1/\varepsilon$	Average number of days before infectious

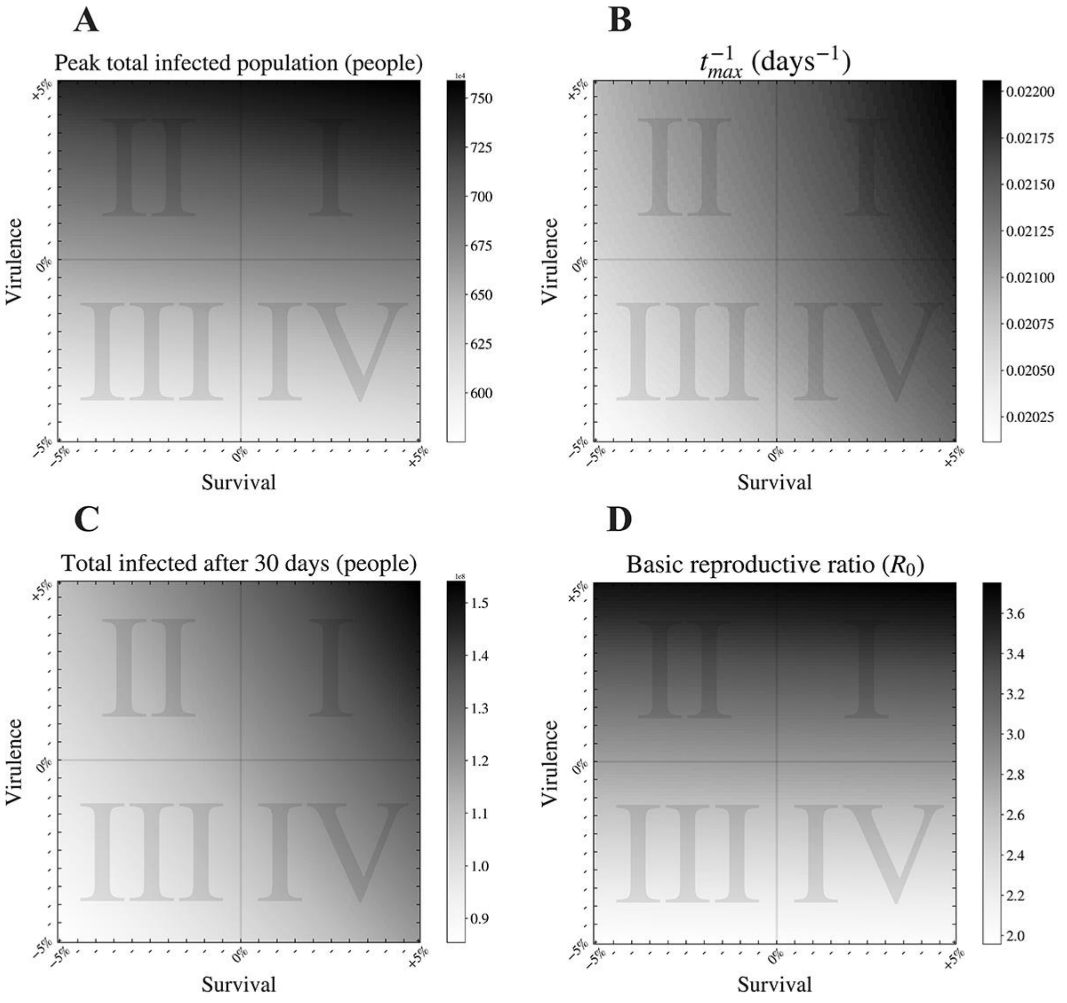

Figure S1. Alternative virulence definition: The impact of varying virus virulence and survival assessed on four key epidemic metrics. As in the main text Figure 4, here we present heatmaps expressing the change in (**A**) the number of infected individuals (asymptomatic and symptomatic) at the epidemic peak, (**B**) the rate at which the epidemic peak is reached, t_{max}^{-1}, (**C**) the total infected population after 30 days, and (**D**) the basic reproductive ratio (R_0) when virulence and survival are modulated by ±- 5% within the.

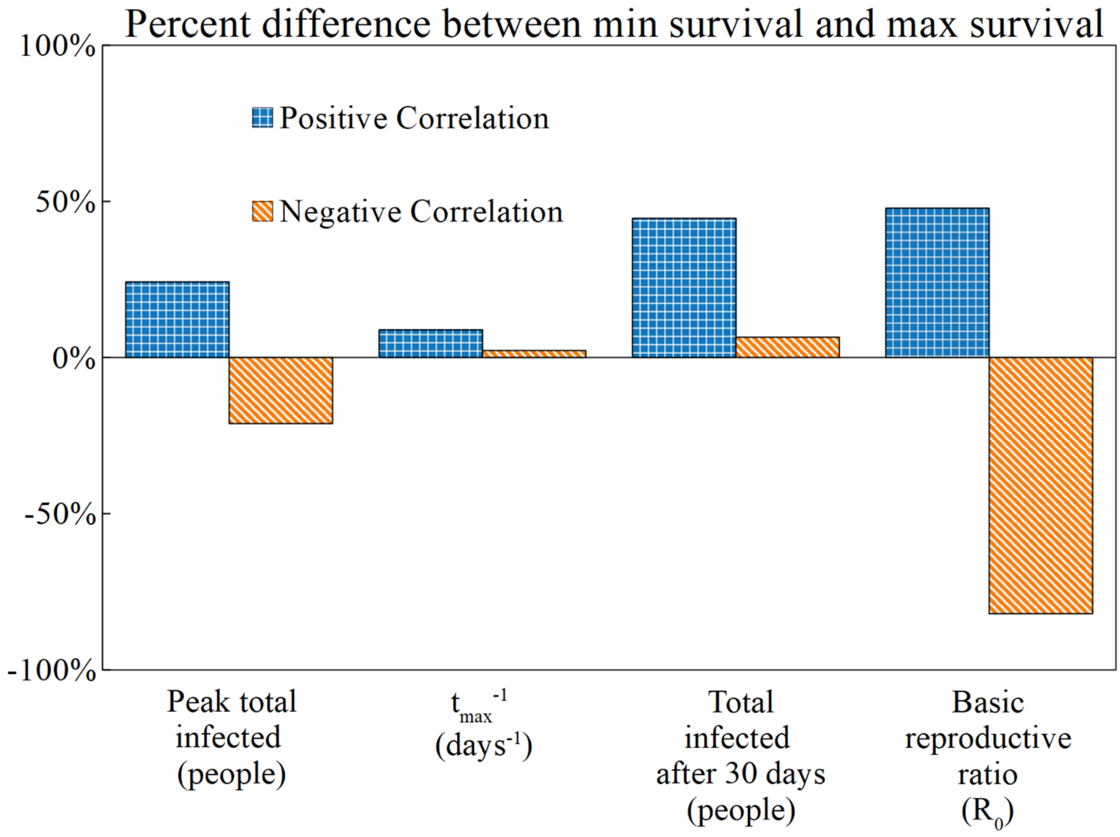

Figure S2. Alternative virulence definition: The percent change in SEAIR-W outbreak metrics as survival increases from −5% to +5% for different virulence-survival relationships (positive correlation and negative correlation). As in the main text Figure 6, for each metric analyzed we present the percent difference between the minimum and maximum survival values given the two hypotheses tested: (i) positive correlation between survival and virulence) comparing low virulence/low survival to high virulence/high survival) and (ii) negative correlation (high virulence/low survival to high survival/low virulence). The bars here correspond to the values (percent) in the third columns of Table 5 and Table 6, which denote the differences between the minimum and maximum values.

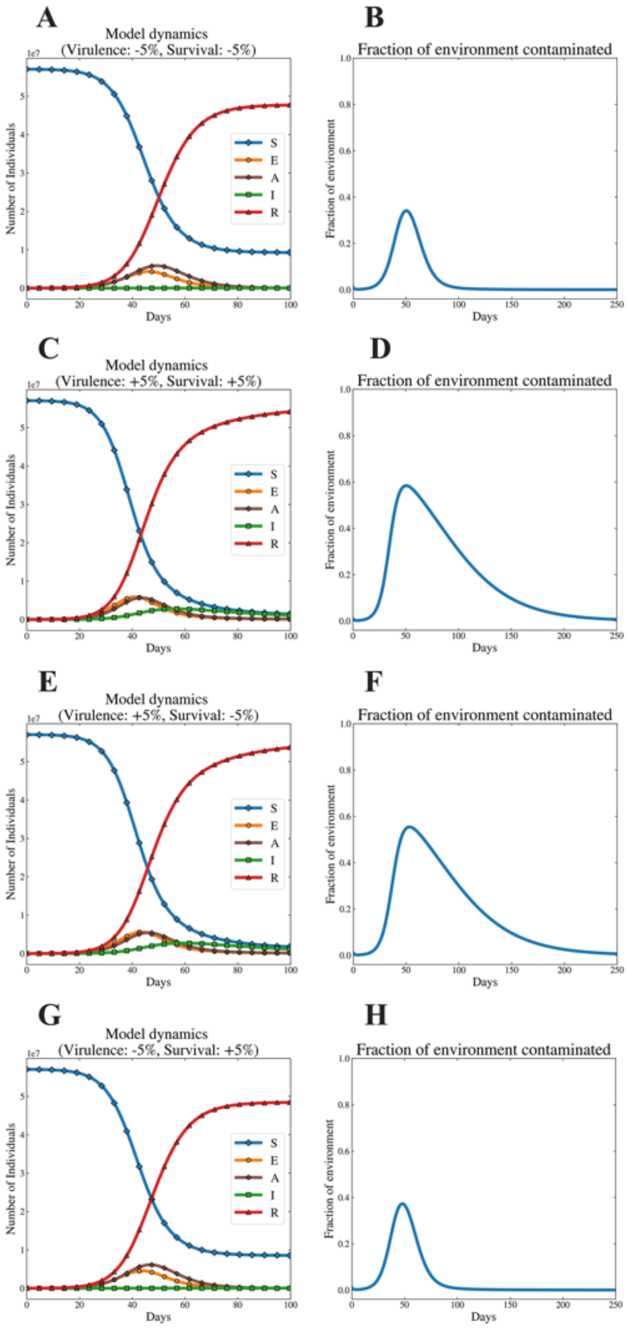

Figure S3. Alternative virulence definition: Virus outbreak dynamics for the extreme values of virulence and free-living survival considered for the two hypotheses. As in the main text Figure 7, here we observe the dynamics of disease corresponding to the extreme values presented in Tables 5 and 6. Subfigures **A,C,E,G** depict disease dynamics, and **B,D,F,H** the dynamics of contaminated environments. Figures 7A–D correspond to the parameter values considered for the Curse of the Pharaoh, while Figures 7E–H correspond to the Goldilocks hypothesis.

Table S3. Alternative virulence definition: Comparing epidemic metrics under low survival/low virulence versus high survival/high virulence scenarios. As in the main text Table 5, for each metric analyzed, these are the heatmap values for the bottom left (at "coordinates" (Vir, Sur) → (−5% ,−5%)) and top right (at "coordinates" (Vir, Sur) → (+5%, +5%)) corners.

positive correlation			
Epidemic Metric	min virulence, min survival	max virulence, max survival	% difference between min survival and max survival
Peak total infected (people)	5.75×10^6	7.59×10^6	24.24%
T_{max-1} (days^{-1})	2.01×10^2	2.21×10^2	8.81%
Total infected after 30 days (people)	8.54×10^7	1.54×10^8	44.59%
Basic reproductive ratio (*R0*)	1.96	3.75	47.83%

Table S4. Alternative virulence definition: Comparing epidemic metrics under low survival/low virulence versus high survival/high virulence scenarios (as in the negative correlation scenario). For each metric analysed, these are the global heatmap values for the top left (at "coordinates" (Vir, Sur) → (+5%, −5%)) and bottom right corners (at "coordinates" (Vir, Sur) → (−5%, +5%)).

negative correlation			
Epidemic Metric	max virulence, min survival	min virulence, max survival	% difference between min survival and max survival
Peak total infected (people)	7.35×10^6	6.07×10^6	−21.13%
T_{max-1} (days^{-1})	2.09×10^{-2}	2.13×10^{-2}	2.28%
Total infected after 30 days (people)	1.11×10^8	1.18×10^8	6.53%
Basic reproductive ratio (R0)	3.64	2.00	−82.04%

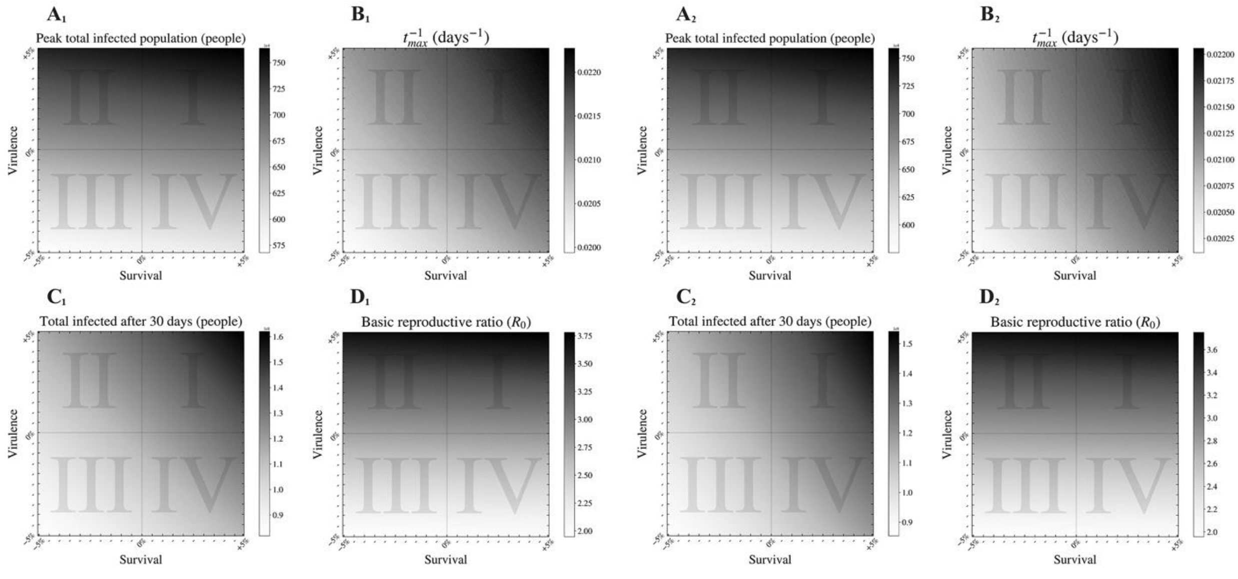

Figure S4. Side by side comparison of the main text virulence definition (A1 → D1) and alternative definition of virulence (A2 → D2).

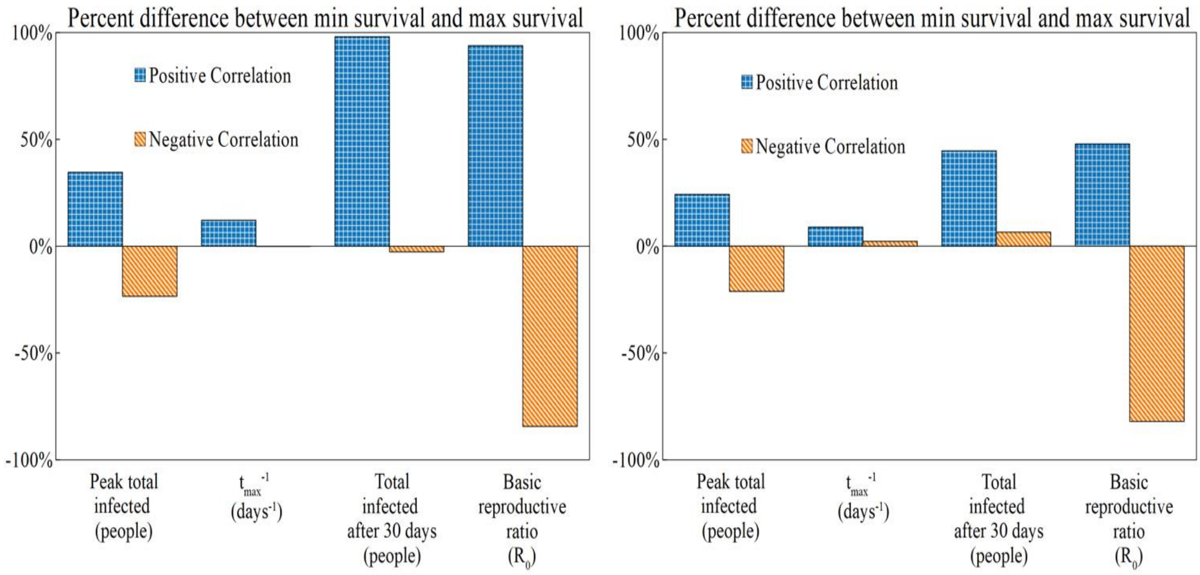

Figure S5. Side by side comparison of the main text virulence definition (left) and alternative definition of virulence (right).

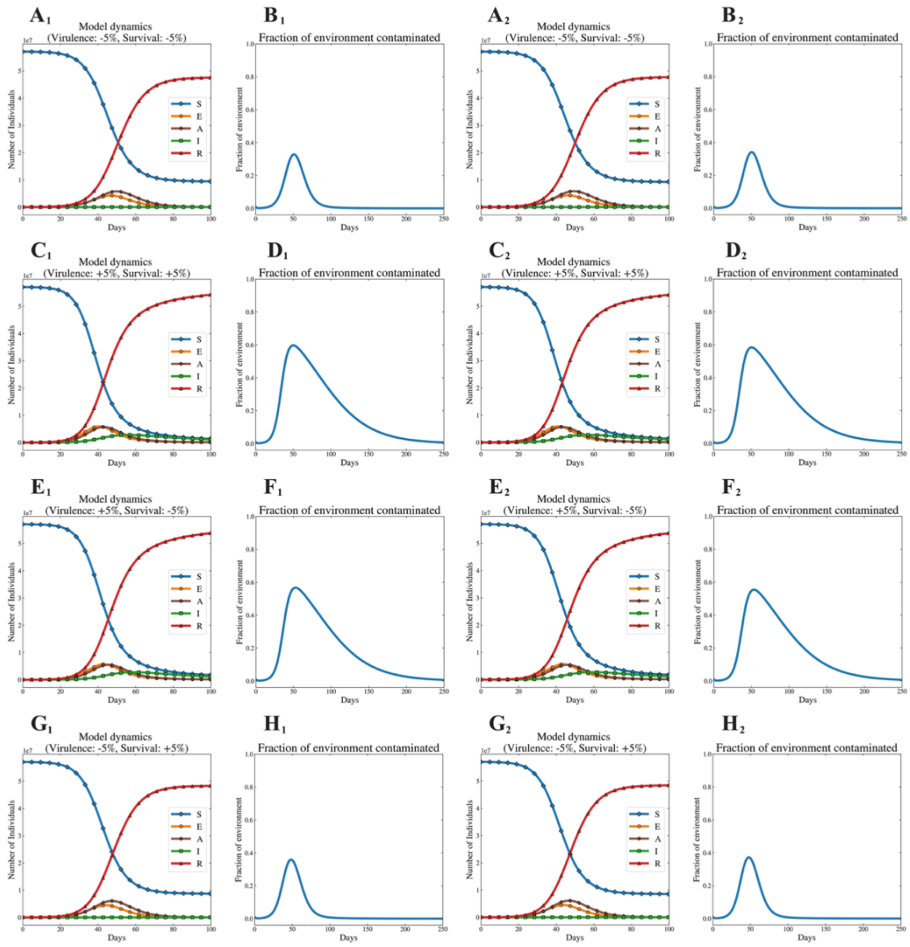

Figure S6. Side by side comparison of the main text definition (**A1** → **H1**) and supplemental definition of virulence (**A2** → **H2**).

We note that for the details on the derivations of the basic reproductive ratio, please see Ogbunugafor et al. 2020 (22). Briefly, the final R_0 form can be observed by following methods as described in prior studies (Diekmann et al., 2009, see main text reference 35). It can be defined by the maximum eigenvalue of the next-generation matrix *G*:

$$G = \begin{pmatrix} R_p & R_{ep} \\ R_{pe} & 0 \end{pmatrix} \quad \text{(S1)}$$

This matrix describes the amount by which the infected populations are scaled from one infection generation to the next (35). In this case, we can consider two infectious components: hosts and the contaminated fraction of the environment. This simplifies *G* to a 2 x 2 matrix. As explained in more detail in Diekmann et al. 2009 (35), this framing preserves the maximum eigenvalue of the epidemic. Another option would be for the modeler to complete the calculation using the full 4 x 4 version of *G*, accounting for all infected compartments (E, I_A, I_S, & W).

The *G* in equation S1 includes the three subcomponents of the R_0 discussed, which is described in Ogbunugafor et al. 2020. Each terms represents an R_0 factor from *a* to *b* where *a* and *b* represent hosts or the environment, making the 0-0 component of *G* the R_0 of host-host direct transmission, the 0-1 component the R_0 applying to rate of transmission from the environment to hosts, the 1-0 component the R_0 of hosts to the environment, and 1-1 component the R_0 of environments to the environment, which is zero in this case (the environment cannot infect itself). In this scenario, the vector of inputs is the column vector given by (infected hosts at time t, contaminated fraction of environment at time t). This can be verified by solving for the maximum eigenvalue of *G*. We reiterate that this calculation, and a fuller explanation, is outlined in Ogbunugafor et al. 2020 (22).

Supplementary References

1. Roser, M.; Ritchie, H.; Ortiz-Ospina, E.; Hasell, J. Coronavirus Disease (COVID-19)—Statistics and Research. Our World Data. 2020. Available online: https://ourworldindata.org/coronavirus (accessed on June 15, 2020).
2. European Centre for Disease Prevention and Control (ECDC). COVID-19 Geographical Distribution Worldwide. 2020. Available online: https://www.ecdc.europa.eu/en/publications-data/download-todays-data-geographic-distribution-covid-19-cases-worldwide (accessed on June 15, 2020).
3. Guan, W.-J.; Ni, Z.-Y.; Hu, Y.; Liang, W.; Ou, C.-Q.; He, J.; Liu, L.; Shan, H.; Lei, C.; Hui, D.S.C.; et al. Clinical characteristics of 2019 novel coronavirus infection in China. *MedRxiv* **2020**, doi:10.1101/2020.02.06.20020974.
4. Wang, W.; Tang, J.; Wei, F. Updated understanding of the outbreak of 2019 novel coronavirus (2019-nCoV) in Wuhan, China. *J. Med Virol.* **2020**, *92*, 441–447, doi:10.1002/jmv.25689.
5. World Health Organization. Report of the WHO-China Joint Mission on Coronavirus Disease 2019 (COVID-19). 2020. Available online: https://www.who.int/docs/default-source/coronaviruse/who-china-joint-mission-on-covid-19-final-report.pdf (accessed on June 15, 2020).
6. Ferguson, N.M.; Laydon, D.; Nedjati-Gilani, G.; Imai, N.; Ainslie, K.; Baguelin, M.; Bhatia, S.; Boonyasiri, A.; Cucunubá, Z.; Cuomo-Danneburg, G . Report 9: Impact of non-pharmaceutical interventions (NPIs) to reduce COVID19 mortality and healthcare demand. *Imp. Coll. Lond.* **2020**, doi:10.25561/77482.
7. Van Doremalen, N.; Bushmaker, T.; Morris, D.H.; Holbrook, M.G.; Gamble, A.; Williamson, B.N.; Tamin, A.; Harcourt, J.L.; Thornburg, N.J.; Gerber, S.I.; et al. Aerosol and surface stability of SARS-CoV-2 as compared with SARS-CoV-1. *N. Engl. J. Med.* **2020**, doi:10.1056/NEJMc2004973.
